# The Role of Pharmacists in Identifying and Preventing Drug-Related Problems in PCOS Management

**DOI:** 10.3390/pharmacy13040095

**Published:** 2025-07-11

**Authors:** Hristina Lebanova, Vesselina Yanachkova, Svetoslav Stoev

**Affiliations:** 1 Department of Pharmaceutical Sciences and Social Pharmacy, Faculty of Pharmacy, Medical University-Pleven, 5800 Pleven, Bulgaria; svetoslav.stoev@mu-pleven.bg; 2 Research Institute, Medical University-Pleven, 5800 Pleven, Bulgaria; v_ess@abv.bg; 3 Department of Endocrinology, Specialized Hospital for Active Treatment of Obstetrics and Gynecology Dr. Shterev, 1330 Sofia, Bulgaria

**Keywords:** polycystic ovary syndrome (PCOS), pharmaceutical care, drug-related problems (DRPs), pharmacist intervention, women’s health, clinical pharmacy

## Abstract

Polycystic ovary syndrome (PCOS) is an endocrine disorder prevalent in women of reproductive age, often requiring complex pharmacological management. The heterogeneity of the syndrome and the use of on- and off-label therapeutic agents—ranging from insulin sensitizers and ovulation inducers to oral contraceptives and herbal supplements—pose significant challenges, including adverse effects, drug interactions, and poor adherence. This narrative review explores the role of pharmacists in identifying and mitigating drug-related problems (DRPs) associated with PCOS therapy. Through thematic synthesis of the current literature, the study highlights common DRPs such as suboptimal drug selection, inappropriate dosing, prolonged therapy duration, and treatment-related safety concerns. It underscores the value of pharmacists’ interventions in enhancing medication adherence, optimizing therapeutic regimens, providing patient education, and monitoring adverse events. A structured, patient-level pharmaceutical care model is proposed, emphasizing personalized assessment, interdisciplinary collaboration, and continuous follow-up. The integration of clinical pharmacists into PCOS care teams has the potential to improve treatment effectiveness, patient satisfaction, and long-term health outcomes. Pharmacists’ contributions are especially critical given the widespread use of off-label therapies and supplements with variable evidence of benefit. Tailored pharmaceutical care can thus bridge the existing gaps in PCOS management and enhance the quality of life for the affected individuals.

## 1. Introduction

Polycystic ovary syndrome is the most prevalent endocrine disorder in women of reproductive age, with a prevalence ranging from 6% to 21%, contingent upon various studies, populations, and diagnostic criteria employed [1,2,3]. The pathophysiological causes of polycystic ovary syndrome are intricate and yet not fully understood. Environmental, genetic, and epigenetic variables significantly influence its etiology. It is widely acknowledged that hyperandrogenemia underlies the reproductive and metabolic disorders associated with the syndrome. Hyperandrogenism is a primary criterion for diagnosing the illness. The Rotterdam criteria, along with those established by the Androgen Excess Society and the National Institute of Health (NIH), constitute the primary points for diagnosing PCOS [3,4,5]. The primary diagnostic signs for the syndrome, as per these recommendations, are hyperandrogenemia, ovulatory dysfunction, and polycystic ovarian morphology evaluated with ultrasound examination [3,4,5].

In addition to hyperandrogenemia, polycystic ovary syndrome is characterized by hormonal disorders such as hyperinsulinemia, hyperprolactinemia, and elevated levels of 17-OH-progesterone, along with frequent fluctuations in carbohydrate and lipid metabolism [3,4,5]. Between 30% and 80% of women with PCOS are classified as overweight or obese [6,7]. The interplay of hormonal disorders and the emergence of Metabolic Syndrome components in these women over time, including subfertility or infertility, may correlate with the onset of type 2 diabetes, hypertension, dyslipidaemia, and increased cardiovascular risk [6,7,8,9].

PCOS is classified into distinct phenotypes—A, B, C, and D—based on differences in its presentation. Phenotype A (most prevalent) encompasses all patients exhibiting ovarian dysfunction, hyperandrogenism, and polycystic ovarian morphology; phenotype B pertains to patients with solely hyperandrogenemia and ovarian dysfunction; phenotype C includes individuals with hyperandrogenemia and polycystic ovarian morphology; and phenotype D describes patients with ovarian dysfunction and ultrasound-confirmed polycystic morphology [10,11,12].

Empirical methods for treatment, commonly used in clinical settings and based on established practices, often result in inadequate therapeutic efficacy and poor patient compliance.

Polycystic ovary syndrome lacks a clearly defined etiology and exhibits significant heterogeneity. The primary objective in the pharmacological management of individuals with PCOS is to restore regular ovulatory cycles, achieve weight reduction when appropriate, and address hyperandrogenism. Currently, there are no medications specifically indicated to treat PCOS [13,14]. A personalized therapeutic approach in PCOS is often lacking due to the absence of a definitive etiological factor and variability among ethnicity, age, and other factors. The current guidelines primarily target individual symptoms rather than the condition as a whole [15,16].

Pharmacological treatment may be associated with various adverse effects, interactions, and adherence challenges, with the likelihood of these issues intensifying as the quantity of pharmaceuticals administered increases. The polypharmacy employed in individuals with PCOS, including diverse supplements, hormonal agents, insulin sensitizers, and dopamine agonists, is a contributing factor to drug-related problems in this group of women. According to the Pharmaceutical Care Network Europe, a drug-related problem is an event or scenario related to medication therapy that either actually or potentially disrupts expected health results [17]. Drug-related problems (DRPs) result in avoidable adverse health consequences. Pharmaceutical care is a crucial approach for recognizing and addressing drug-related problems (DRPs). Pharmacists are integral members of healthcare teams, and their use of pharmaceutical care is a crucial approach for recognizing and addressing drug-related problems [17].

The effective management of PCOS and the prevention of therapy-related complications require a multidisciplinary approach. This includes thorough symptom evaluation, careful consideration of medication quality and cost, assessment of drug interactions, analysis of hormonal and metabolic effects, and strategies to ensure optimal patient compliance and adherence.

The integration of a pharmacist into a multidisciplinary care team managing polycystic ovary syndrome (PCOS), alongside the implementation of targeted and comprehensive pharmaceutical interventions, has the potential to significantly enhance symptom control, optimize clinical outcomes, improve quality of life, and increase patient satisfaction compared to conventional treatment approaches [18,19,20,21,22].

This study aims to critically examine drug-related problems associated with the treatment of polycystic ovary syndrome (PCOS) by synthesizing current evidence from the scientific literature and identifying potential pharmaceutical interventions that pharmacists may employ to enhance the safety, effectiveness, and individualization of pharmacotherapy in this patient population.

## 2. Materials and Methods

This study adopts a narrative review approach to explore drug-related problems in the pharmacological management of polycystic ovary syndrome (PCOS) and to identify potential pharmaceutical interventions that may be implemented by clinical pharmacists. The narrative review format was chosen to allow for a comprehensive and interpretative synthesis of findings across diverse study designs and clinical contexts [23].

A literature search was conducted using databases including PubMed, Scopus, and Google Scholar to identify relevant publications in English. Search terms included combinations of keywords such as “polycystic ovary syndrome,” “PCOS,” “drug-related problems,” “pharmacist intervention,” “pharmaceutical care,” and “clinical pharmacy.” Articles were selected based on their relevance to the pharmacotherapy of PCOS and the identification or discussion of pharmacist-led interventions or medication-related challenges in this patient population. Sources included 52 peer-reviewed original research, reviews, clinical guidelines, and position papers. The collected literature was analyzed thematically to identify common types of drug-related problems (e.g., adverse effects, non-adherence, and therapeutic duplication) and pharmacist contributions, such as medication counseling, therapy optimization, and monitoring. The findings were synthesized to outline practical implications for clinical pharmacy practice in the context of PCOS care.

## 3. Results

### 3.1. Clinical Management Strategies

PCOS is a multifaceted condition that affects multiple organ systems and is characterized by severe metabolic and reproductive manifestations. Treatment should be individualized based on the patient’s specific clinical presentation and the reproductive intentions of the affected woman [24,25,26,27,28,29].

The management of PCOS often involves on- and off-label pharmacological treatments aimed at controlling symptoms and preventing complications associated with the condition (Table 1). For optimal outcomes, it is recommended not only to implement pharmacotherapy but also to adopt an integrated approach, which includes a comprehensive lifestyle modification. This approach encompasses a balanced diet, physical activity, and the inclusion of alternative therapies such as dietary supplements, nutraceuticals, and/or plant-based products with traditional use [30].

The most commonly used drugs are clomiphene citrate, letrozole, metformin, gonadotropic hormones, anti-obesity agents (pioglitazone and semaglutide), and contraceptives (Table 1). Clomiphene citrate (CC) is the most common medication for inducing ovulation in women with polycystic ovary syndrome (PCOS). It is a selective estrogen receptor modulator. CC acts as an antagonist of estrogen receptors in the hypothalamus, leading to an increased synthesis of gonadotropin-releasing hormone (GnRH). In turn, GnRH stimulates higher secretion of follicle-stimulating hormone (FSH) from the pituitary gland. As a result, folliculogenesis and follicular growth are stimulated [40]. The long half-life of the drug (approximately two weeks) leads to long-term adverse effects on the development of the endometrium and cervical mucus [41].

Letrozole is an aromatase inhibitor that acts by reversibly inhibiting the aromatization of androgens into estrogen. As a result, by inhibiting the negative feedback of estrogen on the hypothalamus, it increases the secretion of follicle-stimulating hormone (FSH) from the pituitary gland [42]. Moreover, letrozole is associated with fewer adverse effects due to its higher elimination rate and shorter half-life. However, letrozole is not approved for the treatment of infertility and is still used off-label. Nevertheless, letrozole is considered appropriate for use in women with PCOS who wish to conceive due to its advantages over other ovulation induction medications, and it serves as an important alternative in cases of insufficient response to clomiphene [24,30,43].

Metformin improves insulin sensitivity, reduces insulin levels, and decreases androgen levels [44,45]. The most common side effects of metformin include nausea, abdominal pain, diarrhea, and flatulence, which can contribute to low compliance and unsatisfactory adherence to the therapeutic plan [46]. These adverse effects can be mitigated by utilizing extended-release metformin tablets or by administering the medicine with meals [47].

Gonadotropins are often used for inducing ovulation in women with PCOS who have not ovulated or failed to conceive while undergoing clomiphene citrate therapy [26]. Gonadotropins are hormones naturally produced by the pituitary gland and are used as second-line agents for ovulation induction when there is no response to clomiphene citrate. They work by stimulating the growth and development of follicles in the ovaries [48]. Highly purified FSH (follicle-stimulating hormone) derived from urine, recombinant FSH, and highly purified human menopausal gonadotropin are the primary gonadotropin-containing products used in assisted reproduction therapy [48,49]. The most common drawbacks of these agents include an increased risk of ovarian hyperstimulation syndrome (OHSS) and multiple pregnancies [50].

The standard first-line therapeutic approach for the long-term management of polycystic ovary syndrome involves the use of combined oral contraceptives (COCs) and lifestyle modifications [26]. Combined oral contraceptives containing low doses of estrogen and various types of progestin help improve hyperandrogenism and offer additional benefits, such as reducing the risk of endometrial cancer [32,51]. However, there are concerns regarding the potential cardiometabolic risks associated with these agents. COCs increase the risk of venous thromboembolism (VTE), which is related to both the estrogen dose and the type of progestin used [51]. International guidelines for polycystic ovary syndrome (PCOS), endorsed by ESHRE/ASRM, recommend adherence to the World Health Organization (WHO) guidelines regarding the relative and absolute contraindications for the use of combined oral contraceptives [3].

When used as a treatment, the most notable side effects of spironolactone include reduced androgen levels, improved hirsutism, and acne [52]. The primary mechanism of action involves blocking androgen receptors, partially inhibiting adrenal steroidogenesis, and blocking 5-alpha reductase, thereby increasing the levels of SHBG (sex hormone-binding globulin) [53]. Unlike metformin, spironolactone requires careful dose titration to minimize the risk of side effects. Therefore, spironolactone is recommended to be used in combination with an oral contraceptive to prevent unintended exposure during pregnancy and the associated feminization of male fetuses, a known teratogenic effect. Other side effects associated with high doses include hyperkalemia and menstrual irregularities [54,55].

Myo-inositol (MI) and D-chiro-inositol (DCI) are the two most commonly used forms of inositol. It has been shown that myo-inositol, when administered as a dietary supplement to women with polycystic ovary syndrome (PCOS), improves insulin sensitivity and menstrual regularity while causing fewer gastrointestinal complaints compared to metformin [56]. However, the Cochrane systematic review did not find a promising role for inositol in 1472 women with subfertile polycystic ovary syndrome [57]. Several authors have investigated the effect of D-chiro-inositol on endocrine, metabolic, and reproductive parameters in patients with polycystic ovary syndrome. In a study involving a daily dose of 600 mg DCI administered for 68 weeks to women with PCOS and a BMI of 20.0–24.4 kg/m^2^, a reduction in insulin and free testosterone levels was observed, alongside a decrease in systolic blood pressure, diastolic blood pressure, and serum triglycerides. DCI was associated with a higher ovulation rate, although the difference was not statistically significant. The addition of D-chiro-inositol helped regulate the menstrual cycle [58,59].

Glucagon-like peptide-1 (GLP-1) receptor agonists and other anti-obesity pharmacotherapies are widely acknowledged as supplementary alternatives for weight management in women with polycystic ovarian syndrome (PCOS) when lifestyle modifications prove inadequate. The 2023 International PCOS Guideline suggests that medicines such as liraglutide, semaglutide, and orlistat may be utilized in people with elevated weight, adhering to general obesity treatment guidelines and in conjunction with organized lifestyle assistance [32]. GLP-1 receptor agonists exhibit considerable effectiveness in facilitating weight reduction and enhancing metabolic parameters pertinent to the pathophysiology of PCOS, including insulin resistance. Nonetheless, their administration necessitates meticulous patient selection. Pharmacist counseling should include potential gastrointestinal adverse effects, careful dose escalation to improve tolerance, and the necessity of reliable contraception when pregnancy is possible due to insufficient safety data during gestation. These pharmaceutical treatments must be integrated into a personalized, multidisciplinary care plan, including proper patient education [37].

### 3.2. Herbal Products

Some products containing plant extracts with biologically active compounds (BACs) can be used either individually or in combination to alleviate risk factors associated with polycystic ovary syndrome. Among the plants that have been shown to contain BACs with potential properties for controlling symptoms and/or risk factors of PCOS are *Bauhinia variegata*, beneficial in hormonal imbalance; *Phyllanthus emblica*, *Terminalia bellirica*, *Terminalia chebula*, and *Commiphora wightii*, which are used to regulate hormones; *Cinnamon cassia*, with documented antioxidant effects; *Tribulus terrestris*, which improves reproductive dysfunction; *Hypericum perforatum*, which regulates depression; *Commiphora myrrha*, which prevents menorrhagia; *Nigella sativa*, which controls cholesterol; *Saraca asoca*, which has estrogenic effects; *Asparagus racemosus*, which promotes folliculogenesis; *Tinospora cordifolia*, which regulates menstrual flow; and *Ocimum sanctum*, which has antioxidant properties [60]. It is hypothesized that optimizing the diet through herbal extracts may benefit women with PCOS given their generally good tolerability and presumed safety profile.

### 3.3. Lifestyle Changes

Lifestyle modification is a component of the holistic approach in the treatment of polycystic ovary syndrome (PCOS), complementing pharmacological interventions [61]. The main recommendations include advice on increasing physical activity tailored to the individual characteristics of the woman, dietary adjustments, and weight reduction [61,62]. Optimization of the diet also includes recommendations for the intake of nutraceuticals and dietary supplements aimed at supplementing certain micronutrients and minerals, as well as enhancing insulin sensitivity [63]. Reduction in stress exposure, harmful environmental factors, and the consumption of alcohol and tobacco are also key objectives in the optimized lifestyle for women with PCOS [58,64,65,66].

### 3.4. Drug-Related Problems

Table 2 provides a summary of the most commonly observed drug-related problems in patients with PCOS.

### 3.5. Pharmacists’ Interventions

A key role of the clinical pharmacist in the PCOS pharmacotherapy is accurately identifying the condition, assessing symptom severity, and evaluating the overall health based on clinical manifestations. Proper identification of the need for referral to a specialist, alongside essential interventions such as medication review, dosage adjustments, and deprescribing, constitutes key pharmaceutical practices in the management of women with polycystic ovary syndrome [22,70].

A structured approach to the delivery of pharmaceutical care for patients with PCOS, incorporating a designated protocol for both initial and follow-up consultation, is outlined in Table 3. Some of the potential interventions implemented by pharmacists to ensure rational pharmacotherapy include the following:

- Providing patient education on lifestyle modifications, including physical activity and dietary adjustments, to enhance therapeutic outcomes [71];
- Contributing to the optimal selection of the therapeutic approach, dosage, and specific pharmacological agents based on the patient’s individual characteristics and clinical profile [70,71];
- Pharmacist interventions in the self-management of PCOS using over-the-counter (OTC) products [71,72];

For pharmaceutical care to be optimal, it is necessary that the following conditions are satisfied:

- Patients and healthcare professionals can identify and accurately interpret the impact of PCOS on quality of life, particularly when assessed through the lens of each unique case.
- There is a partnership between healthcare professionals involved in the treatment process of PCOS;
- Individual differences, preferences, and modulating or exacerbating factors are understood;
- All metabolic, reproductive, and psychological characteristics of PCOS are taken into account;
- Awareness, an optimal lifestyle, and emotional well-being are important in managing PCOS [73,74];
- The indigenous population and high-risk ethnic groups are considered.

**Table 3:** Structured approach for the delivery of pharmacological care in patients with PCOS, implementing a designated protocol for both initial and follow-up patient consultations.

FIRST VISIT		
Step	Focus	Pharmacist Intervention
1. Evaluation of patient’s comprehensive health condition	Patient history: menstrual cycle, weight fluctuations, acne, hirsutism, familial propensity, and any other pertinent health issues for risk assessment Pharmacotherapy: current/previous medicines, oral contraceptives, and food supplements Lifestyle: diet, physical activity, and smoking Biochemistry results if available Sufficient minimum data required for the pharmacoeconomic evaluation of the recommended therapy in the specific case/patient	Medication and lifestyle review Document anamnesis and treatment history Selection of potential risk factors and comorbidities of particular relevance to the interaction with PCOS disease and therapy
2. Identification of drug-related problems and patient complaints	Presence of adverse effects and/or interactions Suboptimal therapeutic effectiveness Poor patient compliance/medication adherence Deficiencies in understanding of the condition/therapy	An interview with the patient to identify barriers to adherence Screening for adverse effects; utilization of validated adherence evaluation tools
3. Pharmaceutical interpretation and prioritization	Identify unreported drug-related problems through the application of common strategies for therapeutic optimization, including medication review, analysis of dispensing history, and patient medication reconciliation. Pharmacotherapeutic issues (inadequate dose, drug interactions, or lack of effect) Non-pharmacological factors (unrealistic expectations or poor information)	Medication review; assessment for drug–drug, drug–food, drug–supplement, and drug–condition interactions; risk-based judgment to prioritize issues and drug-related problems; and physician referral or engagement with the prescriber as necessary.
4. Education and counseling	Mechanisms of action of typical medicines (inositol, metformin, combined oral contraceptives, and spironolactone) Explanation of therapeutic goals and possible outcomes Advice on nutrition, exercise, and monitoring of weight and menstrual cycle	Customized patient education; design and implementation of different tools, incl. visual aids or informational brochures to enhance patient knowledge and comprehension; and provision of tools/methods for patient self-regulation and healthful practices for condition/therapy management
**FOLLOW-UP VISIT**		
**Step**	**Focus**	**Pharmacist intervention**
A. Monitor the condition and treatment effect	Changes in symptoms and menstrual cycle Emergence of new adverse reactions Biochemical parameters (if available) such as androgens, FSH, LH, and insulin resistance markers—HOMA-IR, fasting glucose, lipid profile, vitamin B12, and liver enzymes	Reassessment of condition/treatment, ADR reporting if applicable Laboratory values (androgens, LH, FSH, fasting glucose, insulin resistance markers, Vit. B 12 values, and liver enzymes) reassessment and interpretation (if applicable) Recommendation for B12 supplementation when applicable
B. Evaluate progress toward goals	Dynamics in weight, cycle regularity, symptoms (hirsutism, acne); Assessment of the expected therapeutic response	Medication review Evaluation of achieved treatment objectives
C. Adjust therapy/re-apply pharmacists’ interventions (if necessary)	Recommendations to the treating physician to modify sub-optimal doses/medicines Replacement or supplementation with other therapies (e.g., inositols, vitamins, and antiandrogens)	Referral to physician/specialist Deprescribing Dose reduction/dose treatment Guide of nutraceuticals use (by prescribing/recommendation of specific products)
D. Maintain subject motivation	Overcoming barriers to adherence Reinforcing positive results Ongoing patient support through education Patient education to self-assess treatment goals/ADRs	Educational interventions, self-assessment tools provision
Chronology of the pharmaceutical care process A→B→C→D		

Abbreviations used: LH—luteinizing hormone; FSH—follicle-stimulating hormone; HOMA-IR—Homeostatic Model Assessment for Insulin Resistance.

## 4. Discussion

Polycystic ovary syndrome is a multifactorial condition that remains underappreciated in terms of its potential impact on public health, particularly regarding its influence on reproductive health. Furthermore, epidemiological assessments of PCOS prevalence are severely compromised due to local variations in diagnostic criteria and the challenge of differentiating it from other metabolic and non-endocrine disorders. PCOS, in addition to being a distinct clinical entity, also serves as a risk factor for the onset or exacerbation of additional diseases within the cardiovascular, endocrine, and neurological spectra. For patients with PCOS, pharmacists can play a crucial role in optimizing treatment outcomes. Pharmacists should primarily be aware that a specific treatment for the syndrome has yet to be conclusively established. The principal role of both community and clinical pharmacists within the multidisciplinary approach to the management of patients with PCOS is to identify both potential and existing drug-related problems. Furthermore, the pharmacist must possess comprehensive knowledge regarding the condition (including prevention, diagnosis, and management) to deliver interventions that contribute to the overall improvement of patient quality of life through the promotion of rational pharmacotherapy. A model algorithm for the pharmacist’s approach to patients with PCOS has been provided in Table 3 based on the aforementioned results. The organized methodology (Table 3) illustrates how a pharmacist might incorporate medication evaluations, adherence assessments, and patient education into standard care practices. By carefully identifying DRPs—such as prolonged clomiphene use or inappropriate letrozole timing, therapy-associated B12 deficiency, or suboptimal effectiveness of nutraceuticals—pharmacists have the potential to decrease risks of treatment failure, adverse effects, and patient dissatisfaction.

The current research highlights the intricate and diverse pharmacotherapy of PCOS, frequently further confounded by the off-label usage of therapeutic approaches. These findings underline the critical role of the pharmacist in identifying drug-related problems (DRPs) and improving patient outcomes through personalized therapies and pharmacist interventions that align standard treatment with the individual profile of a specific patient. PCOS can be managed with medications such as metformin, thiazolidinediones, as well as oral contraceptives, letrozole, spironolactone, GLP-1 agonists, and others. Moreover, pharmacists have a key role in the proper identification of the condition and referral of the patient, recognizing the syndrome based on symptoms that overlap with those of other conditions (e.g., type 2 diabetes). It is important to understand the potential risks and ensure that they are communicated to patients, whether these risks arise from PCOS itself or adverse reactions related to the therapy for the condition.

Non-pharmacological approaches, such as lifestyle changes in patients with PCOS, can play a pivotal role in managing the condition. Furthermore, our hypothesis regarding the applicability of pharmacist intervention for optimum dietary regimens is consistent with the existing literature and published evidence, which suggests that dietary modifications proposed by pharmacists constitute a relevant intervention to consider [72]. Additionally, the use of dietary supplements, vitamins, minerals, and plant-based products is frequently practiced by women with PCOS, regardless of the scientific evidence supporting the health claims made [75,76].

Although certain patients favor herbal remedies and nutraceuticals, the existing research is inconclusive. The Cochrane review referenced in our study demonstrates inconclusive advantages of inositol, notwithstanding its prevalent application [57]. This discrepancy indicates that pharmacists should rigorously evaluate supplement assertions and convey realistic expectations to patients.

In this context, pharmacists serve as the final barrier to the use of inappropriate medications, drug interactions, and exposure to potentially ineffective products. The pharmacist is a key communicator of risks, both associated with the condition itself and with the specific products being used. This function may be executed successfully through pharmaceutical interventions, including individualized nutraceutical product recommendations based on patient requirements and characteristics, dose reduction, or even deprescribing to mitigate risks or avoid interactions.

Pharmacists can act as gatekeepers against improper self-medication with off-label or unverified herbal items, which are often inadequately controlled in numerous areas.

Patients taking oral contraceptives should be advised on the correct usage, the importance of adherence to therapy, and all possible side effects, such as breast tenderness, spotting, weight gain, and fluid retention [77]. Pharmacist interventions, including dosage reduction in oral contraceptives or recommendations for transitioning to low-estrogen or progestin-only contraceptives in collaboration with the prescriber, are optimal strategies for improving patient outcomes.

Our analysis indicated that although pharmacological interventions such as metformin, clomiphene citrate, letrozole, and hormonal contraceptives are fundamental, considerable obstacles endure concerning adherence, adverse drug reactions, and the necessity of patient education for optimal therapeutic outcomes.

Our results align with recent systematic reviews demonstrating that the gastrointestinal side effects of metformin contribute to dropout rates of up to 20% [78]. The established prolonged half-life of clomiphene corroborates previous cautions regarding alterations in endometrial and cervical mucus, underscoring the necessity for individualized treatment strategies.

Pharmacist intervention involving the formulation of a tailored administration regimen, which includes the recommendation to take the medication before meals and to incrementally increase the dosage from once daily by 500 mg each week to alleviate adverse gastrointestinal effects, has also been acknowledged as a strategy for enhancing patient adherence in a review by Attia et al. [79]. The anticipated outcome of metformin therapy suggests that B12 deficiency, resulting from reduced absorption due to metformin, may necessitate pharmacist intervention for the initiation of B12 supplementation.

Furthermore, pharmacists play a crucial role in identifying potential drug interactions between PCOS medications, such as those caused by the concurrent use of certain antibiotics with oral contraceptives [80]. Through patient education about PCOS, advice on potential long-term effects should be provided, and treatment adherence should be encouraged to reduce the risks of metabolic syndrome, type 2 diabetes, and endometrial cancer [81].

Several limitations of the existing discourse on the pharmaceutical care approach in individuals with PCOS should be acknowledged. This study is constrained by the absence of empirical evidence substantiating the efficacy of the suggested strategy across varied healthcare environments. Furthermore, discrepancies in diagnostic criteria among groups may restrict the applicability of certain guidelines and, consequently, some proposed pharmacist interventions. Consequently, future research should concentrate on prospective assessments of pharmacist-led PCOS care programs, encompassing cost-effectiveness analyses and patient-reported outcome measures. Moreover, meticulously structured clinical trials are essential to elucidate the function of inositol, D-chiro-inositol, and herbal extracts in the treatment of PCOS.

## 5. Conclusions

The management of polycystic ovary syndrome, a common endocrine condition among women of reproductive age, requires a multimodal approach. Pharmacological treatments such as oral contraceptives, antiandrogens, insulin sensitizers, and ovulation-inducing agents can help hormonal imbalances, irregular menstruation, reproductive challenges, and metabolic issues like insulin resistance and dyslipidemia. These therapies may present drug-related problems that require pharmacist intervention. Pharmaceutical services can assist PCOS patients in symptom management, attainment of reproductive objectives, and enhancement of metabolic health through evidence-based drugs, personalized care, and interdisciplinary teamwork.

## Figures and Tables

**Table 1:** Most common medications in the management of polycystic ovary syndrome.

Condition	Treatment	Dosage	Possible ADRs	Reference
**Insulin Resistance**	Metformin	A total of 500 mg (initial dose) with increases of 500 mg within 1–2 weeks, and employing extended-release formulations may reduce side effects and improve adherence	Nausea, flatulence, and vitamin B12 deficiency	Lashen [31], Teede [32]
**Irregular Menstrual Cycle**	Oral Contraceptives (OCPs)	20–35 µg Low-estrogen dose OCPs (e.g., EE 20 µg)	Weight gain, VTE, spotting, and breast tenderness	Nader [33], Domecq, et al. [34], Teede [32]
**Hirsutism**	Cyproterone acetate (monotherapy)	10 mg	Headache, breast tenderness	Nader [33], Teede [32]
	Ethinylestradiol (in combination)	20–50 µg		
**Infertility**	Clomiphene citrate, Letrozol	Clomiphene citrate: 50–150 mg for 5 days; Letrozole: 2.5–5 mg/day (days 3–7 of menstrual cycle)	Nausea and mood changes Multiple pregnancies; hot flashes	Nader [33], Practice Committee of the American Society for Reproductive Medicine [35]
**Hyperandrogenism**	Spironolactone, Flutamide, Finasteride	Spironolactone: 25–100 mg Flutamide: 500 mg + OCP	Spironolactone: fatigue, irregular cycle (at high doses), hyperkalemia Finasteride: hepatotoxicity Flutamide: hepatotoxicity	Nader [33], Domecq, et al. [34], Teede [32]
**Obesity**	Pioglitazone, GLP-1-agonists, Orlistat	Pioglitazone: 15–30 mg/daily; GLP-1-agonists: Semaglutide 0.25^−1^ mg once weekly; Liraglutide 0.6–1.8 mg once daily, Orlistat 120 mg twice a day	Pioglitazone: mild peripheral edema and muscle cramping; GLP-1-agonists: gastrointestinal symptoms (nausea, diarrhea, vomiting, constipation, abdominal pain, or dyspepsia); Orlistat: increased bowel movement, oily stool, oily spotting, stomach pain, and some cases of liver injury	Sangeeta S. [36], Szczesnowicz A., et al. [37], Kumar P., et al. [38], Panda., et al. [39]

Abbreviations: ADR—adverse drug reaction; EE = ethinylestradiol; GLP-1 = glucagon-like peptide-1.

**Table 2:** Drug-related problems in PCOS (coded according to the PCNE Classification for Drug-Related Problems V9.1).

Primary Domain	Cause	Product	Code	Problem	Pharmacists’ Role
Treatment effectiveness	Drug selection (C1)	Inositol & myo-inositol	P1.2	Varying effectiveness of dietary supplements containing inositol and myo-inositol in women with different phenotypes of polycystic ovary syndrome [67,68]	Pharmacists need to possess sufficient skills not only to identify PCOS but also to assess the phenotype of the disease and the individual characteristics of the patients, based on which they can decide on the recommendation for additional intake of myo-inositol
	Treatment duration (C4)	Inositol & myo-inositol	P1.2	Insulin sensitizers such as inositol/myo-inositol, including when combined with folic acid, require prolonged administration for at least three months [69]	Ensuring high levels of patient compliance and adherence
	Drug selection (C1)	Clomiphene citrate	P1.2	Insufficient data on the efficacy of clomiphene citrate in women with polycystic ovary syndrome and clear reproductive intentions to conceive a child [11,35,40]	Providing evidence based on the use of clomiphene citrate in women with PCOS and reproductive intentions
	Drug selection (C1)	Herbal products	P1.1	The controversial data regarding the efficacy of herbal plant extracts in controlling symptoms associated with PCOS [60]	A critical analysis of scientific publications and results from various types of studies is crucial for providing reliable, evidence-based information, both when consulting other healthcare professionals and when directly communicating with patients.
Treatment safety	Drug selection (C1)	Clomiphene citrate	P2.1	The data on the long plasma half-life of clomiphene citrate (approximately two weeks) raise concerns about the potential for long-term, delayed adverse effects on the development of the endometrium and cervical mucus [41]	Effective risk communication is crucial when interpreting data on the benefit–risk ratio associated with the use of these products.
	Drug form (C2) Dose selection (C3)	Metformin	P2.1	Nausea, flatulence, abdominal pain, diarrhea, and bloating are key factors contributing to low compliance and the expected suboptimal adherence to the prescribed therapy [46,47] Metformin is linked to the risk of vitamin B12 insufficiency due to malabsorption resulting from its mode of action.	Selection of a specific medicinal product allows for risk reduction through the use of extended-release metformin tablets. Development of an optimal dosing regimen through gradual dose titration during the initiation of pharmacological therapy. Patient education
	Dose selection (C3)	Gonadotropins	P2.1	Increased risk of ovarian hyperstimulation syndrome and the likelihood of multiple pregnancies [50]	Providing education on proper medication use, ensuring appropriate dosing, monitoring for adverse effects, and counseling patients/healthcare professionals on the importance of adhering to treatment protocols
	Drug selection (C1) Treatment duration (C4)	COCs	P2.1	VTE; potential risk of myocardial infarction, disturbances in glucose metabolism and diabetes, elevated levels of triglycerides, serum cholesterol, and total cholesterol [51]	Educating patients on medication adherence, lifestyle modifications, monitoring health parameters, and collaborating with healthcare providers to optimize treatment and prevent complications.
	Dose selection (C3)	Spironolactone	P2.1	Teratogenic potential and likelihood of feminization of a male fetus in cases of unintended exposure during early pregnancy, as well as the risk of hypokalemia and various menstrual irregularities [54]	Providing patient education on the risks of medication use during pregnancy, ensuring proper contraceptive use, monitoring for adverse effects, and counseling on the importance of early pregnancy screening and avoiding unintended exposure to teratogenic drugs.
	Dose selection (C3)	GLP1-receptor agonists	P2.1	GI adverse effects, nausea, and vomiting	Pharmaceutical interventions encompass a dose titration regimen administered by the pharmacist, along by dietary guidance and education to consume modest quantities and avoid certain food kinds.
	Drug selection (C1)	Finasteride	P2.1	Finasteride may be associated with sexual dysfunction and alterations in mood.	Comprehensive information on the accurate identification of finasteride-related possible adverse drug reactions (ADRs) and the implementation of strategies for patient follow-up.
Other	Drug use process (C6) Patient-related (C7)	Letrozole Clomiphene	P3.1	Letrozole and clomiphene citrate are administered during specific phases of the menstrual cycle to achieve targeted modulation of the hypothalamic-pituitary-ovarian axis and stimulate follicle-stimulating hormone (FSH) secretion [35,40,41,42]. Non-adherence to the prescribed timing may lead to deviation from the therapeutic window, thereby diminishing the pharmacological efficacy and resulting in suboptimal clinical outcomes.	Consultation on optimal dosing regimens
	Drug dispensing (C5)	Letrozole	P3.1	Off-label use	Optimizing the dispensing process

Abbreviations: COC = combined oral contraceptive; VTE = venous thromboembolism; ADR = adverse drug reaction; DRP = drug-related problem.

## Data Availability

The original contributions presented in this study are included in the article. Further inquiries can be directed to the corresponding author.
